# Follicular cells protect *Xenopus* oocyte from abnormal maturation *via* integrin signaling downregulation and *O*-GlcNAcylation control

**DOI:** 10.1016/j.jbc.2023.104950

**Published:** 2023-06-23

**Authors:** Alain Martoriati, Caroline Molinaro, Guillaume Marchand, Ingrid Fliniaux, Matthieu Marin, Jean-François Bodart, Yoshiko Takeda-Uchimura, Tony Lefebvre, Vanessa Dehennaut, Katia Cailliau

**Affiliations:** 1 University Lille, CNRS, UMR 8576-UGSF-Unité de Glycobiologie Structurale et Fonctionnelle, Lille, France; 2 Université de Lille, CNRS, INSERM, CHU Lille, UMR9020-U1277-CANTHER-Cancer Heterogeneity Plasticity and Resistance to Therapies, Lille, France

**Keywords:** *Xenopus laevis*, oocytes, ovarian follicular cells, integrin, Grb7, EGF receptor, OGT, OGA, *O*-GlcNAcylation

## Abstract

*Xenopus* oocytes are encompassed by a layer of follicular cells that contribute to oocyte growth and meiosis in relation to oocyte maturation. However, the effects of the interaction between follicular cells and the oocyte surface on meiotic processes are unclear. Here, we investigated *Xenopus* follicular cell function using oocyte signaling and heterologous-expressing capabilities. We found that oocytes deprotected from their surrounding layer of follicular cells and expressing the epidermal growth factor (EGF) receptor (EGFR) and the Grb7 adaptor undergo accelerated prophase I to metaphase II meiosis progression upon stimulation by EGF. This unusual maturation unravels atypical spindle formation but is rescued by inhibiting integrin β1 or Grb7 binding to the EGFR. In addition, we determined that oocytes surrounded by their follicular cells expressing EGFR–Grb7 exhibit normal meiotic resumption. These oocytes are protected from abnormal meiotic spindle formation through the recruitment of *O*-GlcNAcylated Grb7, and OGT (*O-*GlcNAc transferase), the enzyme responsible for *O*-GlcNAcylation processes, in the integrin β1–EGFR complex. Folliculated oocytes can be forced to adopt an abnormal phenotype and exclusive Grb7 Y338 and Y188 phosphorylation instead of *O*-GlcNAcylation under integrin activation. Furthermore, an *O*-GlcNAcylation increase (by inhibition of *O*-GlcNAcase), the glycosidase that removes O-GlcNAc moieties, or decrease (by inhibition of OGT) amplifies oocyte spindle defects when follicular cells are absent highlighting a control of the meiotic spindle by the OGT–*O*-GlcNAcase duo. In summary, our study provides further insight into the role of the follicular cell layer in oocyte meiosis progression.

In most ovaries of animal species, oocytes are surrounded by a layer or enclosed in a mass of somatic cells (1). In *Xenopus* amphibians, oocytes are covered with a single layer of flat stellate follicle cells, also named follicular cells with extended macrovilli that contact the oocyte surface (2). These ovarian somatic follicular cells exert important functions during oogenesis to acquire competencies for fertilization (3, 4). They support several aspects of oocyte growth, including vitellogenesis and meiosis arrest during maturation (2, 5). If the interactions between the *Xenopus* follicular cells and the oocyte have been abundantly described to explain the vitellus uptake and the vitelline pool formation in the oocyte (2, 6), their function in meiosis progression remains elusive. The disconnection of follicular cells from the oocyte inhibits vitellogenesis and affects oogenesis (7), but no clear evidence supports their role in maintaining the oocyte meiotic arrest during oogenesis. Both oocytes and follicular cells are rich in cytoskeletal structures and integrins embedded in their plasma membranes (5, 8, 9, 10). These components can work in concert to provide bidirectional transmission of mechanical and biochemical signals across the plasma membrane (11). In other species such as mammals, bidirectional communication occurs between the oocyte and the granulosa cells, somatic cells closely associated with the developing oocyte (12, 13, 14). At the time of ovulation, integrins act to separate the oocyte from the granulosa cells (15). In *drosophila*, integrins regulate and maintain a monolayered follicular epithelium around the oocyte throughout oogenesis (16, 17).

Stage VI *Xenopus laevis* oocytes are arrested in prophase I and progress during maturation in metaphase II where they arrest waiting fertilization (18, 19). The maturation of prophase I oocytes can be induced by progesterone (20) or by insulin (21) and insulin growth factor 1 (IGF1) (22) to their respective receptor tyrosine kinase (RTK) expressed in oocytes and follicular cells (3). Oocyte meiotic resumption requires a whole set of post-translational modifications including the phosphorylation/dephosphorylation of major effectors of the cell cycle such as Cdk1 and mitogen-activated protein kinase (MAPK)–Erk2, being the most widely described (23, 24). Nevertheless, an increase in global *O*-GlcNAcylation is also crucial for the oocyte meiotic resumption before activation of the M phase maturating factor (Cdk1/cycle B) and MAPK–Erk2 pathways (25, 26, 27). *Xenopus* oocytes naturally express only one type of RTK: the insulin–IGF1 receptor. However, because of their highly efficient translational machinery, oocytes have been used as a useful heterologous-expressing system (28, 29). Various RTKs have been expressed in oocytes to decipher their specific downstream signaling pathways upon stimulation by the appropriate ligand (30, 31, 32, 33). Among them, the EGF receptor (EGFR), when expressed in oocytes and following its stimulation by EGF, activates canonical signaling pathways involving kinases, phosphatases, and molecular adaptors, which result in maturation (31, 33). Grb7 is an adaptor protein recruited in several RTK complexes (34, 35, 36) and a critical modulator of somatic and cancer cell proliferation (37). Originally identified as an activated-EGFR-binding partner, Grb7 also interacts with cytoplasmic kinases such as focal adhesion kinase (FAK) (38) that generate and amplifies the RTK–integrin nongenomic pathways (35, 39, 40, 41). Moreover, EGFR has been described as pivotal to trigger ovulation in mammals by uncoupling the follicular cells from the oocyte (42, 43).

In an attempt to understand both the function of *Xenopus* follicular cells in oocyte meiosis progression from prophase I to metaphase II and the role played by integrins, we used the translational ability of oocytes to express the EGFR and the Grb7 adaptor. *Xenopus* oocytes deprotected from their follicular cells undergo an abnormally rapid maturation and display a nonanchored abnormal meiotic spindle. The molecular mechanism involves the phosphorylation of Grb7 by FAK in an integrin–EGFR heteromeric complex. *In fine*, we found that follicular cells protect the oocyte through the recruitment of *O-*GlcNAc transferase (OGT) and the subsequent *O*-GlcNAcylation of Grb7 avoiding its phosphorylation by the FAK–integrin signaling axis in response to EGFR activation. This novel unraveled mechanism allows a correct maturation and spindle anchorage to the plasma membrane. In addition, OGT and *O*-GlcNAcase (OGA) correct regulation is also necessary for meiotic maturation.

## Results

### Oocytes unsheathed from their surrounding follicular cells and expressing EGFR and Grb7 display an accelerated maturation

**Figure 1:**
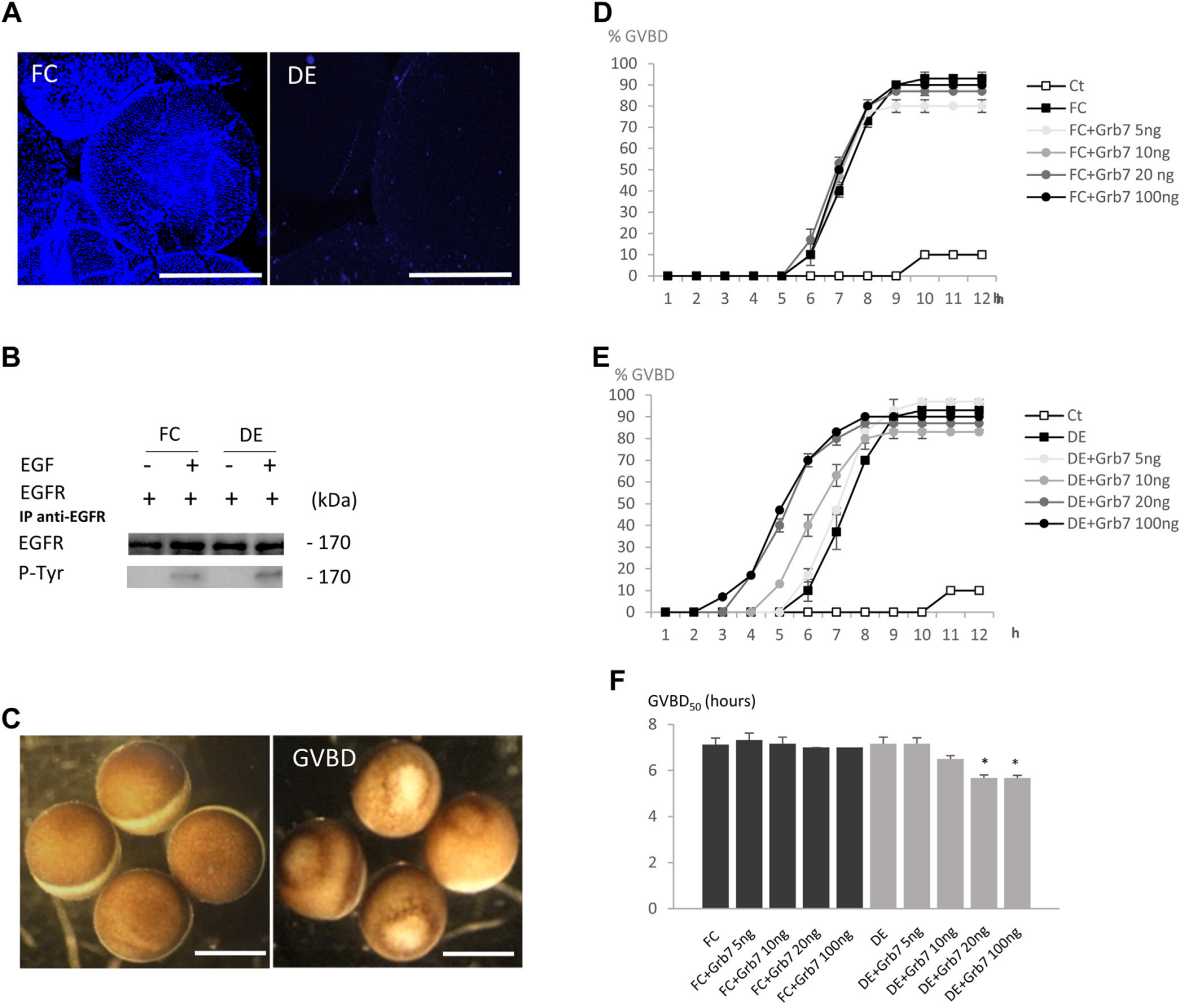
**Grb7 triggers an accelerated germinal vesicle breakdown (GVBD) in oocytes deprotected from their layer of surrounding follicular cells and expressing the epidermal growth factor (EGF) receptor (EGFR).** The human EGFR was expressed in stage VI *Xenopus* oocytes surrounded by follicular cells (FCs) and stage VI oocytes deprotected from their follicular cells (DEs, defolliculated oocytes). One hour before EGF stimulation (5 nM) or not in control (Ct), Grb7 was microinjected in the equatorial region at concentrations of 5, 10, 20, or 100 ng. *A*, Hoechst 33258 staining of the surrounding layer of oocytes with follicular cells (*left*) or defolliculated (*right*); the scale bars represent 1000 μm. *B*, EGFR immunoprecipitations followed by Western blots with anti-EGFR and anti–phospho-tyrosine (P-Tyr). *C*, example of GVBD of stage VI *Xenopus* oocytes; the scale bars represent 1000 μm. *D* and *E*, the GVBD attesting for the progression from prophase I to metaphase II (maturation) was scored 12 h after stimulation by EGF or not (Ct) in FC and DE oocytes. *F*, the GVBD_50_ representing the time required to obtain half of the response of FC and DE oocytes to undergo GVBD was determined. Experiments were performed on 10 to 20 oocytes from three different females. Statistical significance was accepted in DE and FC for ∗*p* < 0.05.

*Xenopus* oocytes were unsheathed from their surrounding follicular cells and injected with RNA encoding the EGFR before nuclei were labeled with Hoechst 33258 (Fig. 1*A*). EGF stimulation of the receptor results in tyrosine autophosphorylation in both cases (Fig. 1*B*). One hour before stimulation with EGF, the adaptor protein Grb7 was microinjected at various concentrations. The occurrence of the maturation, or germinal vesicle breakdown (GVBD), is a faithful witness of prophase I to metaphase II transition. It is characterized by the appearance of a white spot at the animal-pigmented pole and is easily scored in oocytes with or without their surrounding follicular cells (Fig. 1*C*). Without EGF stimulation, neither EGFR expression with or without Grb7 microinjection nor Grb7 alone induces a significant GVBD (respectively 1% ± 0.1%, 10% ± 4%, and 3% ± 0), in oocytes with or without their surrounding follicular cells as previously described (31, 34, 35). Grb7 did not alter EGF-induced maturation when follicular cells were present, whereas it accelerates the GVBD in a dose-dependent manner (20 and 100 ng) when follicular cells were removed from oocytes expressing EGFR (Fig. 1, *D* and *E*). The half-maximal time attesting for a maturation (GVBD_50_) is significantly shorter for unsheathed oocytes expressing EGFR and microinjected with 20 ng or 100 ng of Grb7 or not (respectively 5 h 40 min ± 17 min and 5 h 35 min ± 16 min) compared with oocytes expressing EGFR without Grb7 (7 h 10 min ± 1 7 min) (Fig. 1*F*). GVBD_50_ obtained for folliculated oocytes (FCs) had similar timing whatever experimental conditions were applied.

**Figure 2:**
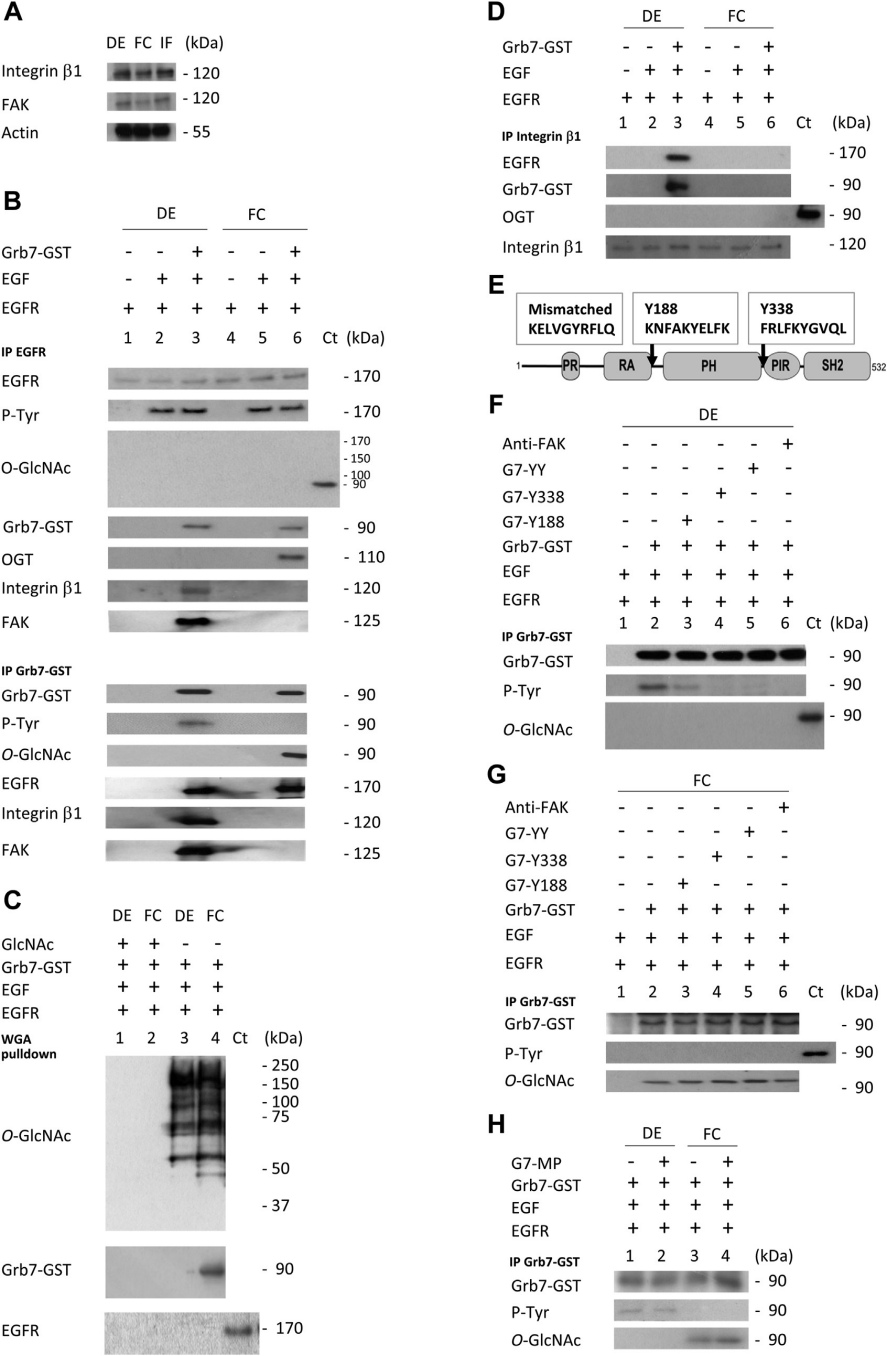
**In oocytes deprotected from their follicular cells, the epidermal growth factor receptor (EGFR)–EGF signaling complex recruits integrin β1, phosphorylated Grb7, but lacks OGT**. Stage VI *Xenopus* oocytes, with surrounding follicular cells (FC) or defolliculated (DE), expressing EGFR were injected with 20 ng of Grb7 or not 1 h before EGF stimulation (5 nM) or left unstimulated. *A*, after protein normalization, Western blots were performed on FC or DE oocytes, and isolated follicular cells (IFs) with anti-integrin β1, anti-FAK, and anti-actin antibodies. *B*, immunoprecipitations were performed with anti-EGFR or anti-GST-Grb7 antibodies, and Western blots were performed with antibodies against EGFR, phospho-tyrosine (P-Tyr), *O*-GlcNAcylation (*O*-GlcNAc), GST (Grb7-GST), *O*-linked *N*-acetylglucosamine (GlcNAc) transferase (OGT), FAK, and integrin β1. Control (Ct) was an immunoprecipitation performed with anti-GST-Grb7 on FC cells expressing EGFR and injected with Grb7. *C*, sWGA pulldowns were performed with or without competition with 0.5 M free GlcNAc and followed by Western blot analysis with anti-*O*-GlcNAcylation (*O*-GlcNAc) or anti-GST-Grb7 antibodies. *D*, after immunoprecipitations with anti-integrin β1, Western blots were performed with anti-EGFR, -GST (Grb7-GST), -OGT, and -integrin β1 antibodies. Ct: control as in *B*. *E*, scheme showing Grb7 domains and competitive Grb7 peptides: proline-rich (PR), RalGEF/AF6, or Ras associated like (RA), Pleckstrin homology (PH), phospho-tyrosine interacting region PIR (BPS, between PH and SH2); and Src homology 2 (SH2). *F*–*H*, Grb7 peptides Y188, Y338, both peptides (G7-YY), control mismatched peptide (G7-MP), or anti-FAK antibodies were microinjected with Grb7 (20 ng), 1 h before EGF stimulation. Ct: control as in *B*. Experiments were performed on 25 oocytes and three different females. FAK, focal adhesion kinase; GST, glutathione-*S*-transferase; sWGA, succinylated wheat germ agglutinin.

We further investigate the possibility of a crosstalk between integrin and EGFR relaying the activated signaling pathways in defolliculated oocytes (DEs). Integrin β1 and FAK are detected in DE, FC, and in isolated follicular cells (Fig. 2*A*). EGFR immunoprecipitations reveal that the discrepancy between DE and FC EGR/Grb7-expressing oocytes is not linked to the receptor phosphorylation state. No difference in the EGFR tyrosine phosphorylation is detected between DE and FC oocytes with 20 ng of Grb7 (Fig. 2*B*). Unstimulated EGFR displays no tyrosine phosphorylation. In addition, Grb7 is immunoprecipitated in the EGFR signaling complex in DE and FC oocytes. Integrin β1 and FAK are only linked to the EGFR complex in DE oocytes (Fig. 2*B*). OGT is only present in the EGFR complex from FC oocytes (Fig. 2*B*). When the same samples are submitted to glutathione-*S*-transferase (GST)-Grb7 pull down, DE oocytes display a tyrosine-phosphorylated form of Grb7, whereas this phosphorylation is not detected in FC oocytes for which Grb7 is conversely *O*-GlcNAcylated (Fig. 2*B*). To ascertain the specificity of the interactions, immunoglobulin G (IgG) controls for immunoprecipitation are shown in Fig. S1. *O*-GlcNAc protein enrichment on succinylated wheat germ agglutinin (sWGA)-agarose beads (44) followed by a Western blot analysis showed Grb7 is present in FC oocytes but not in DE oocytes (Fig. 2*C*). Control realized with 0.5 M free GlcNAc as competitor shows no Grb7 detection (Fig. 2*C*). In addition, integrin coprecipitates with EGFR and Grb7 in deprotected DE oocytes but not in protected FC oocytes (Fig. 2*D*).

To gain better insight into the differences observed in the recruitment of integrin β1 at the level of the EGFR complex and into the discrepancies of post-translational modifications of Grb7, competition experiments were led by using peptides mimicking the major FAK-phosphorylated site in Grb7 and with FAK antibodies. In DE oocytes, both Y188 and Y338 peptides and FAK antibodies (Fig. 2, *E* and *F*) impede the tyrosine phosphorylation of Grb7 in the EGFR signaling complex. Y188 when used alone only reduces the Grb7 phosphorylation state (Fig. 2*F*) showing tyrosine phosphorylation of Grb7 occurs on both sites Y188 and Y338 and requires FAK. In these conditions, no *O*-GlcNAcylation is detected on Grb7. With FC oocytes, competition experiments performed with Y338, Y338 added to Y188, or FAK antibodies could not abolish Grb7 *O*-GlcNAcylation (Fig. 2*G*). Control peptides formed by mismatched sequences flanking the tyrosine residue could not modify Grb7 tyrosine phosphorylation or its *O*-GlcNAcylation (Fig. 2*H*).

### In oocytes protected by their layer of surrounding follicular cells, the interaction of OGT with the EGFR complex and the subsequent Grb7 *O*-GlcNAcylation depends on integrin

**Figure 3:**
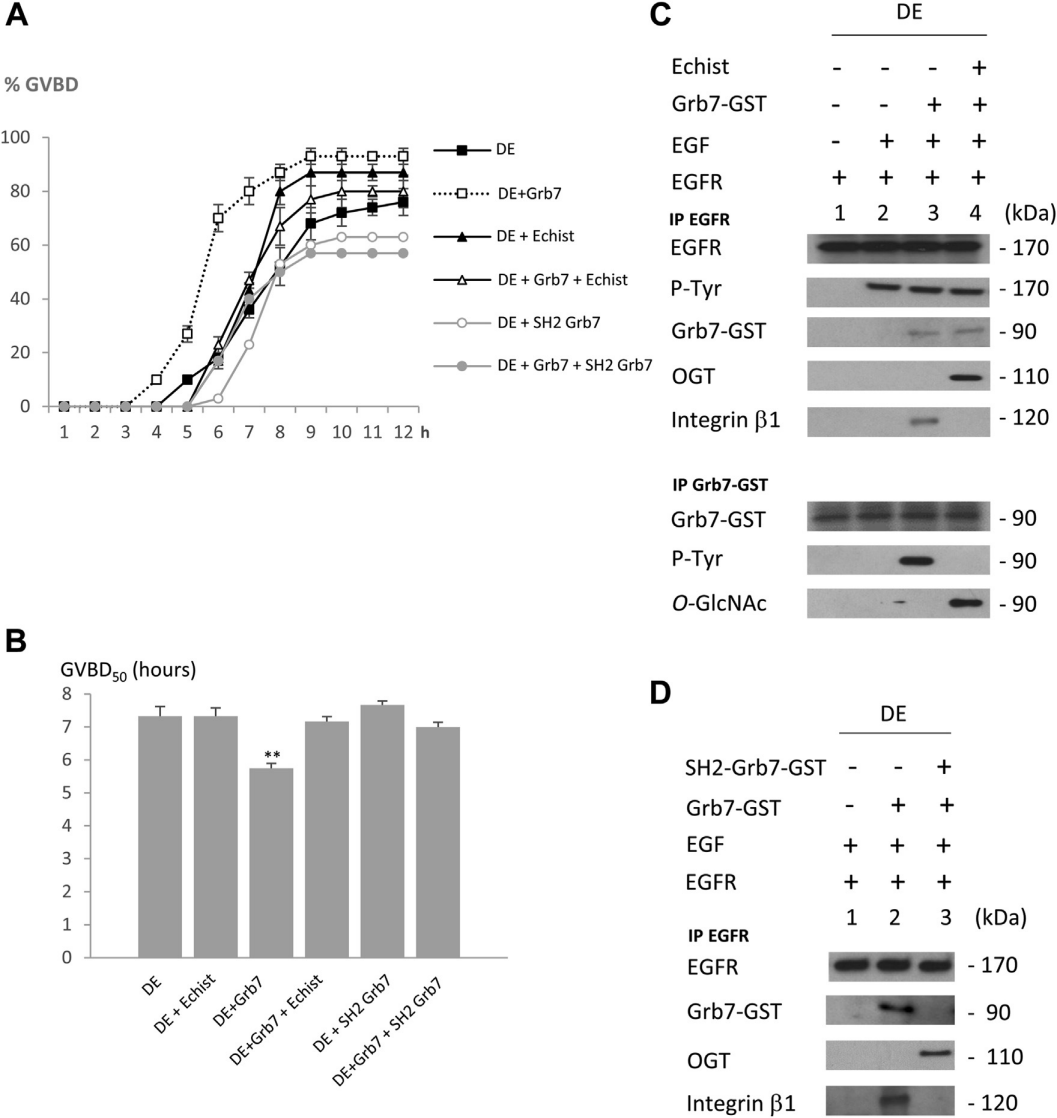
**In defolliculated oocytes (DEs) expressing epidermal growth factor receptor (EGFR)–Grb7, integrin inhibition rescues germinal vesicle breakdown (GVBD)**. Stage VI *Xenopus* oocytes deprotected from follicular cells (D), and expressing EGFR were stimulated or not by EGF (5 nM), 1 h after microinjection with Grb7 (20 ng), with or without SH2 domain of Grb7 60 ng, and before an incubation or not with 500 nM of integrin inhibitor echistatin (Echist) for 12 h. *A*, the GVBD attesting for meiosis progression (maturation) was scored. *B*, the GVBD_50_ representing the time of half-responsive DE oocytes to undergo GVBD was determined. *C* and *D*, immunoprecipitations were performed with anti-EGFR or anti-GST-Grb7 antibodies. Western blots were performed with antibodies against EGFR, phospho-tyrosine (P-Tyr), *O*-GlcNAcylation (*O*-GlcNAc), GST (Grb7-GST), *O*-linked *N*-acetylglucosamine (GlcNAc) transferase (OGT), and integrin β1. Experiments were performed on three different females. Statistical significance against DE was accepted for ∗*p* < 0.05 and ∗∗*p* < 0.01. GST, glutathione-*S*-transferase.

**Figure 4:**
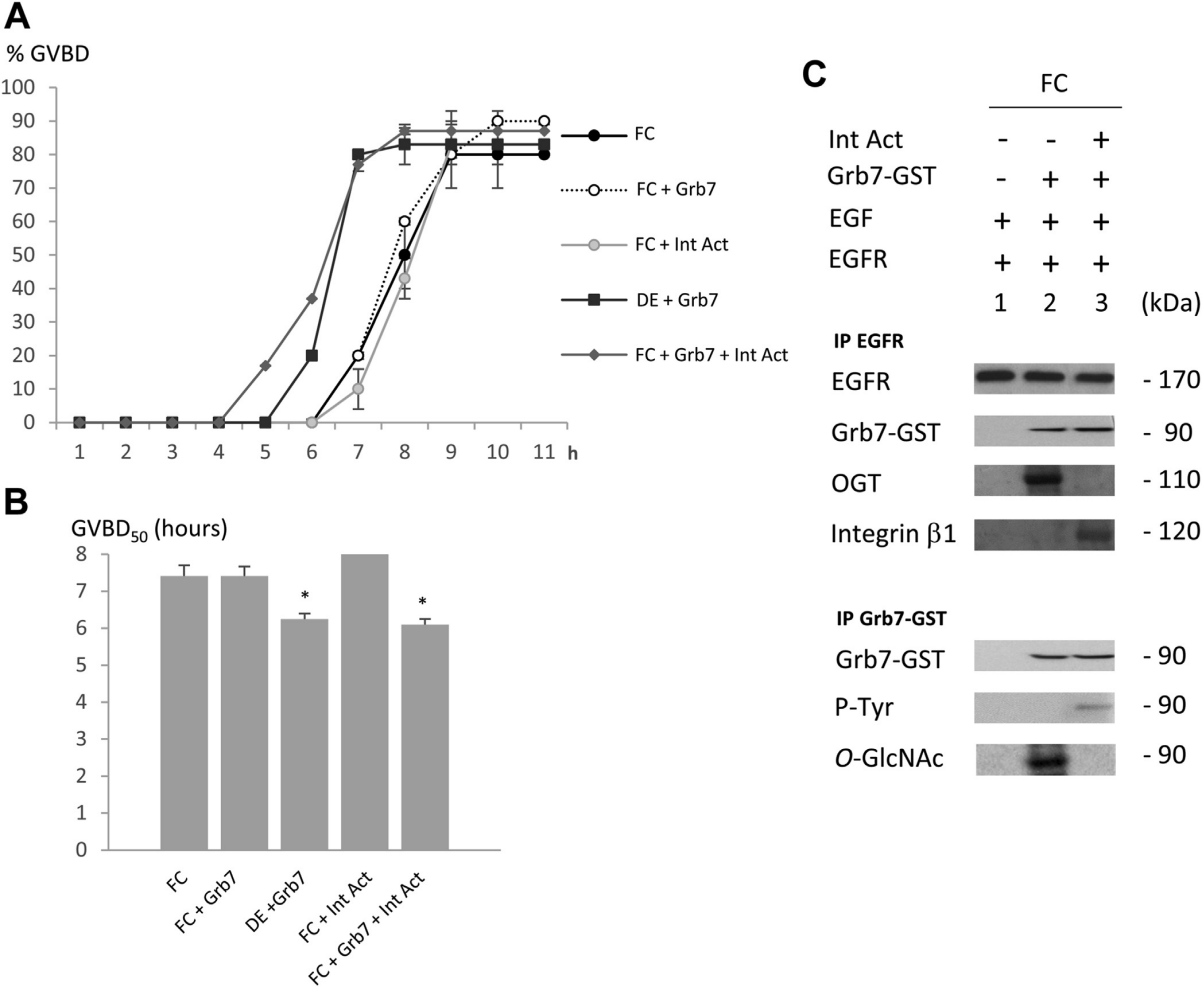
**In folliculated oocytes, integrin activation triggers germinal vesicle breakdown (GVBD) acceleration, and neither OGT nor *O*-GlcNAcylated Grb7 are recruited in the epidermal growth factor receptor (EGFR) complex.** Stage VI *Xenopus* oocytes displaying their follicular cells layer (FC) and expressing EGFR were injected or not 1 h before EGF stimulation (5 nM) with Grb7 (20 ng) and incubated or not with 10 μM of pyrintegrin activator (Int Act) or left unstimulated. *A*, the GVBD attesting for meiosis progression was scored. *B*, the GVBD_50_ (time of half responsive FC oocytes to undergo GVBD) was determined. *C*, immunodetections were performed after immunoprecipitations with anti-EGFR or anti-GST-Grb7 antibodies and followed by Western blots with antibodies against EGFR, GST (Grb7-GST), *O*-linked *N*-acetylglucosamine [GlcNAc] transferase) (OGT), integrin β1, and *O*-GlcNAcylation (*O*-GlcNAc). Experiments were repeated on 20 oocytes and three females. Statistical significance against FC was accepted for ∗*p* < 0.05. GST, glutathione-*S*-transferase.

Incubation with the integrin inhibitor echistatin (Sigma–Aldrich), a member of the disintegrin family, at 500 nM or microinjection of Grb7-competing SH2 domains to avoid Grb7 recruitment to the EGFR complex (60 ng) in DE oocytes significantly restores a classical GVBD_50_ (Fig. 3, *A* and *B*). SH2 domains of Grb7 also reduce the percentage of GVBD obtained compared with controls (Fig. 3*A*). Into the EGFR complex of oocytes incubated with echistatin, EGFR exhibits no changes in its tyrosine phosphorylation status. Grb7 is recruited with OGT in the EGFR complex, whereas integrin β1 is not (Fig. 3*C*). Moreover, with the integrin inhibitor, Grb7 *O*-GlcNAcylation is restored, whereas its tyrosine phosphorylation is lost (Fig. 3*D*). Competition experiments performed with the SH2 domains of Grb7 lead to the dissociation of full-length Grb7 and integrin β1 from the EGFR complex, whereas, in return, OGT is recruited into the complex (Fig. 3*D*). Reverse experiments were performed in FC oocytes expressing EGFR, microinjected with Grb7, and incubated with an integrin activator (pyrintegrin, 10 μM). In these conditions, GVBD_50_ are significantly lower and similar to those obtained for DE oocytes (Fig. 4, *A* and *B*). Early GVBD_50_ is associated with Grb7 and integrin β1 binding and a loss of OGT binding in the EGFR complex. In parallel, we observed that immunoprecipitated Grb7 is phosphorylated on tyrosine and its *O*-GlcNAcylation abrogated (Fig. 4*C*).

### EGFR–integrin crosstalk *via* Grb7 has a deleterious effect on maturation including spindle assembly

**Figure 5:**
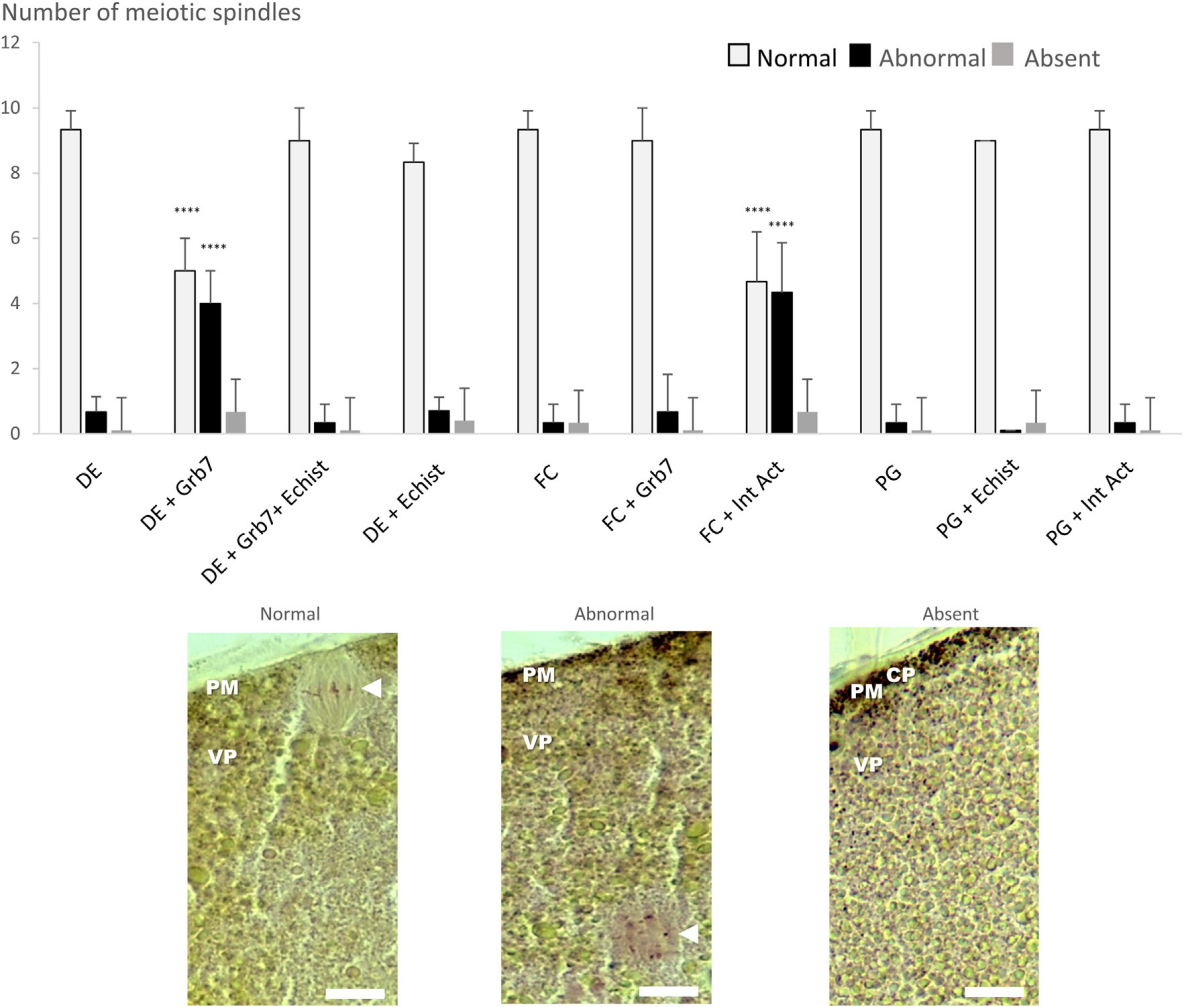
**Abnormal spindle formation occurs in defolliculated epidermal growth factor receptor (EGFR)–Grb7-expressing oocytes that display germinal vesicle breakdown (GVBD) acceleration.***Xenopus* defolliculated oocytes (DEs) and folliculated oocytes (FCs) expressing EGFR were injected or not with Grb7 (20 ng) and incubated or not with 500 nM of integrin inhibitor echistatin (Echist) for DE or with Grb7 (20 ng) with or without 10 μM of pyrintegrin activator (Int Act) for FC, 1 h before EGF stimulation (5 nM); controls were left unstimulated. Naïve oocytes were treated with progesterone (PG). Oocytes were fixed, dehydrated, and paraffin-embedded before sections (7 μm) were stained with nuclear red to detect nuclei and chromosomes and picroindigocarmine to reveal cytoplasmic structures. The normal, abnormal, or absent spindles were scored. Cortical pigment (CP), plasma membrane (PM), and vitelline platelet (VP); the *arrow* points to the chromosomes on the spindle, and the scale bar represents 35 μm. Statistical significance against DE or FC was accepted for ∗∗∗∗*p* < 0.001.

In DE oocytes expressing EGFR–Grb7, the meiotic maturation results in abnormal meiotic spindles that were not anchored to the plasma membrane (45, 46) (Fig. 5). Normal spindles were detected in FC oocytes expressing EGFR–Grb7 and under progesterone stimulation added or not with the integrin inhibitor (echistatin, 500 nM) or activator (pyrintegrin, 10 μM) (Fig. 5). The addition of the integrin inhibitor echistatin to DE oocytes restores normal spindle formation, whereas the integrin activator (pyrintegrin, 10 μM) added to FC oocytes leads to abnormal spindle formation (Fig. 5). Controls performed without Grb7 but with an integrin activator or inhibitor result in normal meiotic spindle formation both in DE and FC oocytes (Fig. 5).

### Favoring or inhibiting *O*-GlcNAcylation in DE/deprotected oocytes modulates abnormal spindle formation induced by the integrin–EGFR–Grb7 complex

**Figure 6:**
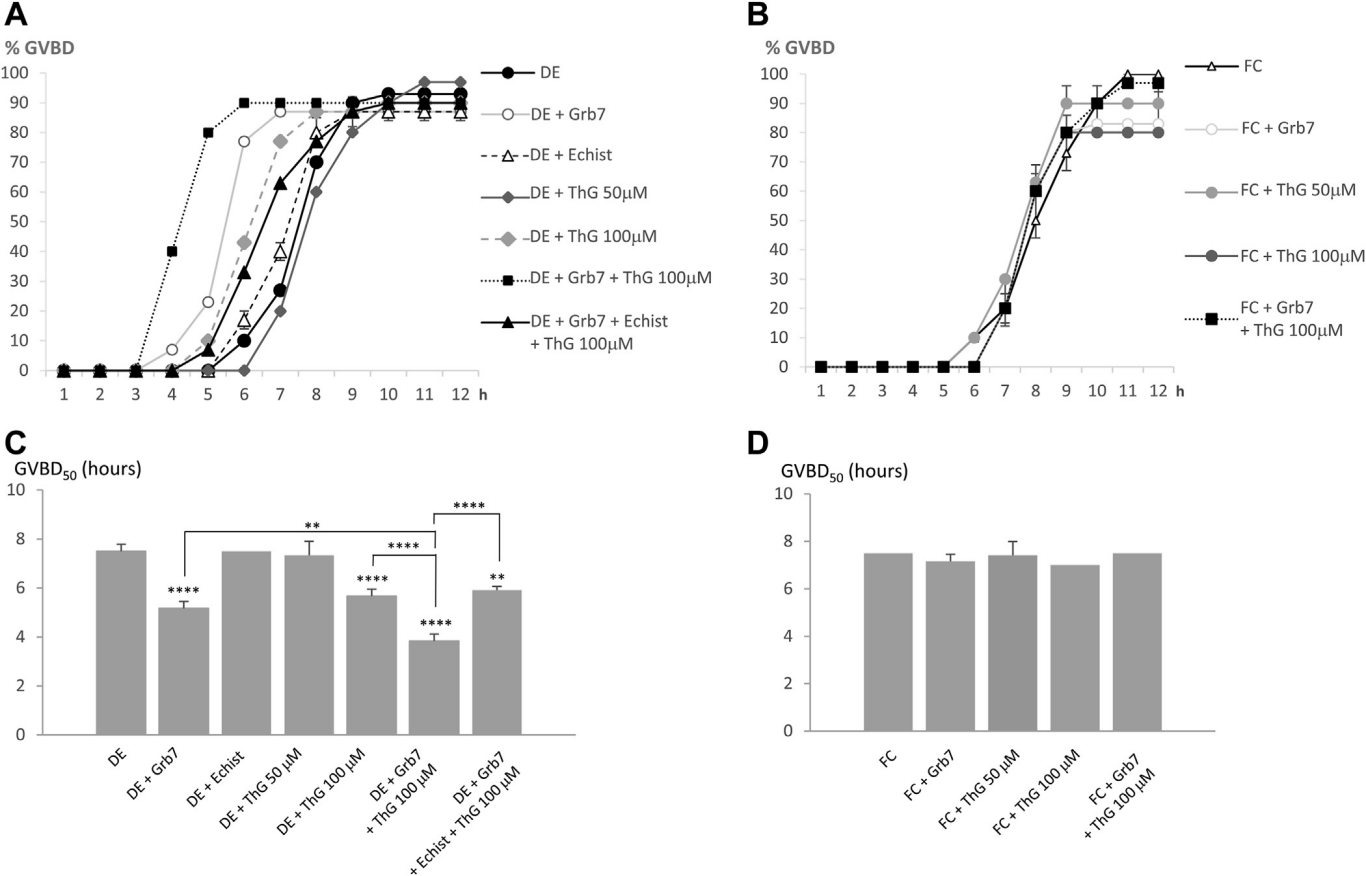
**Favoring *O*-GlcNAcylation by *O*-GlcNAcase (OGA) inhibition lowers GVBD**_**50**_**in defolliculated oocytes (DEs)**. *Xenopus* DE and folliculated oocytes (FCs), expressing epidermal growth factor receptor (EGFR), were microinjected with Grb7 (15 ng) or not, incubated or not with 50 or 100 μM OGA inhibitor Thiamet G (ThG), 1 h before EGF stimulation (5 nM). DE oocytes were submitted to integrin inhibitor echistatin 500 nM (Echist). *A* and *B*, the germinal vesicle breakdowns (GVBDs) were scored. *C* and *D*, GVBD_50_ (time of half-responsive FC oocytes to undergo GVBD) were determined. Results were obtained on 20 oocytes from three different females. Statistical significance was accepted for ∗∗*p* < 0.01 and ∗∗∗∗*p* < 0.001. GVBD_50,_ half-maximal time attesting for a maturation.

**Figure 7:**
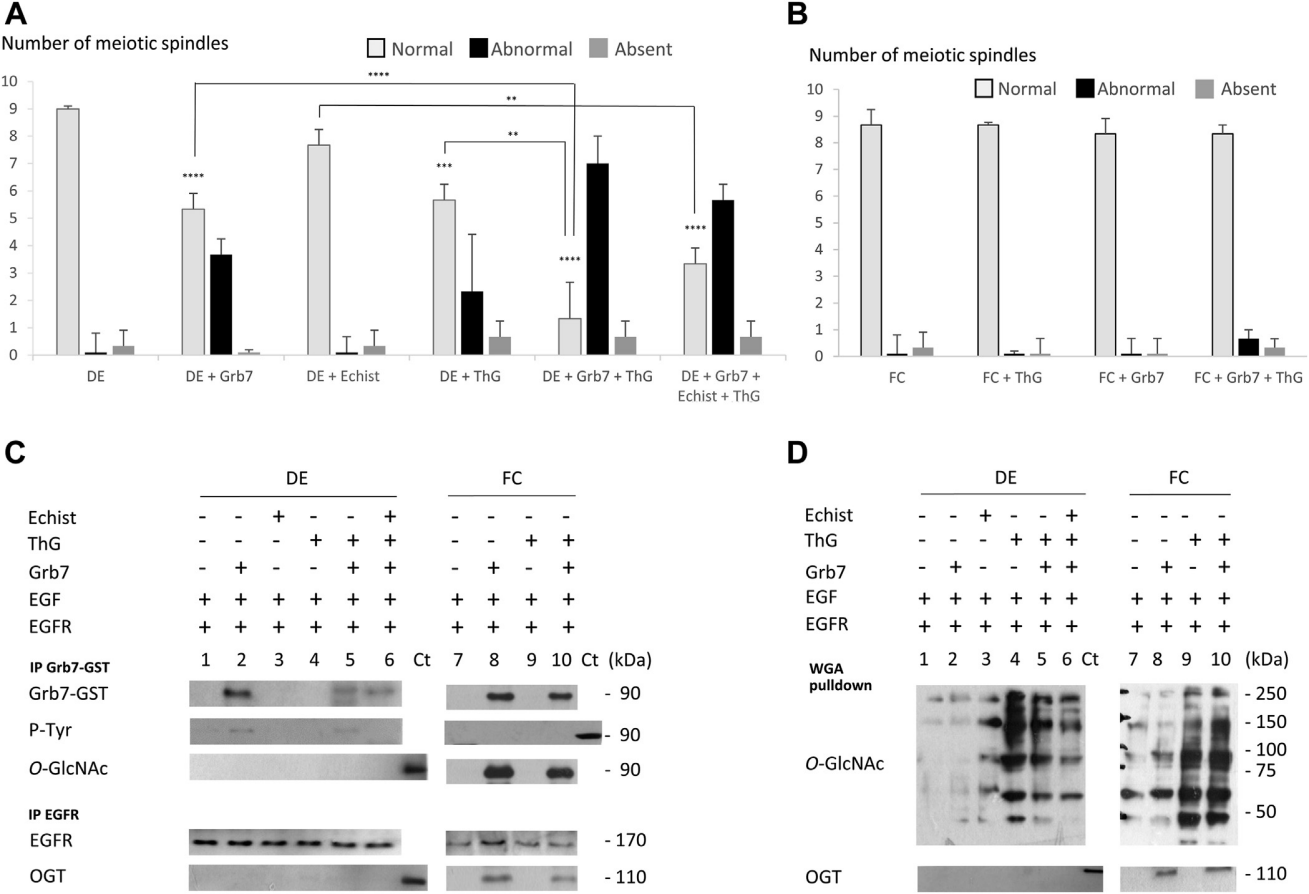
**Favoring *O*-GlcNAcylation by *O*-GlcNAcase (OGA) inhibition increases abnormal spindles in defolliculated oocytes (DEs).***Xenopus* DEs and folliculated oocytes (FCs) expressing epidermal growth factor receptor (EGFR) were microinjected with Grb7 (15 ng) or not, incubated or not with 100 μM OGA inhibitor Thiamet G (ThG), 1 h before epidermal growth factor (EGF) stimulation (5 nM). DEs were submitted to 500 nM of integrin inhibitor echistatin (Echist). *A* and *B*, the normal, abnormal, or absent spindles were scored in oocytes fixed, dehydrated, and paraffin-embedded on sections (7 μm) stained with nuclear red (nuclei and chromosome detection) and picroindigocarmine (cytoplasmic structure detection). *C*, Grb7-GST and EGFR immunoprecipitations were realized and followed by Western blotting with anti-*O*-GlcNAcylation (*O*-GlcNAc), anti-GST (Grb7-GST), and anti-OGT antibodies. *D*, sWGA pulldowns were performed before Western blotting with anti-*O*-GlcNAcylation (*O*-GlcNAc) and anti-OGT antibodies. Results were obtained on 10 to 20 oocytes from three different females. Statistical significance was accepted for ∗∗*p* < 0.01, ∗∗∗*p* < 0.005, and ∗∗∗∗*p* < 0.001. GST, glutathione-*S*-transferase; sWGA, succinylated wheat germ agglutinin.

**Figure 8:**
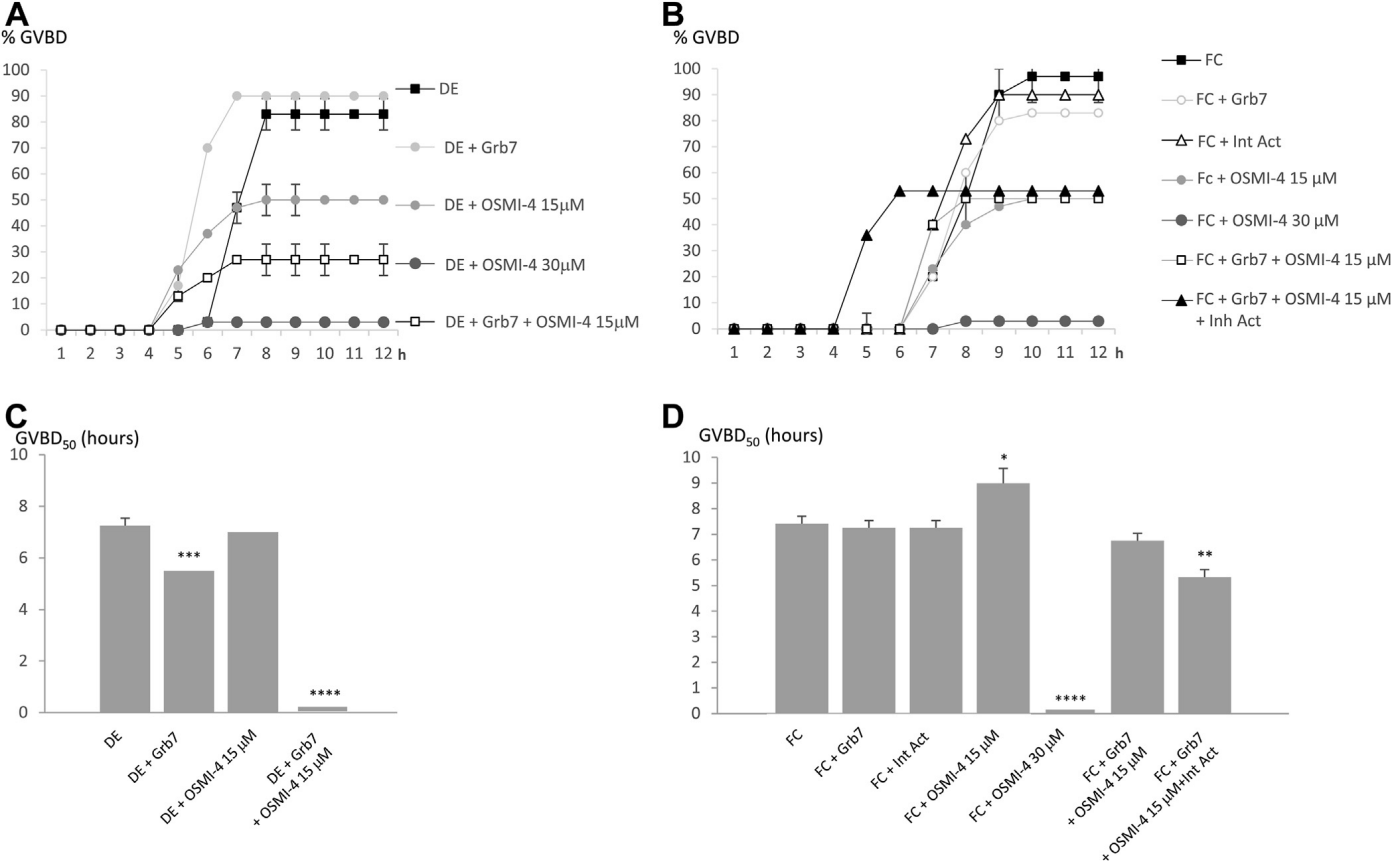
**Inhibition of *O*-GlcNAcylation using *O*-GlcNAc transferase (OGT) inhibitor inhibits oocyte germinal vesicle breakdown (GVBD).** Defolliculated (DE) and folliculated oocytes (FCs) expressing epidermal growth factor (EGF) receptor (EGFR) microinjected with Grb7 (15 ng) or not were incubated or not with 15 and 30 μM OGT inhibitor OSMI-4, 1 h before EGF stimulation (5 nM). FCs were treated or not with 10 μM of pyrintegrin activator (Int Act). *A* and *B*, the GVBDs were scored during 12 h. *C* and *D*, GVBD_50_ (time of half-responsive FC oocytes to undergo GVBD) were determined. Experiments were repeated on 20 oocytes from two to three *Xenopus* females. Statistical significance against DE or FC was accepted for ∗*p* < 0.05, ∗∗*p* < 0.01, ∗∗∗*p* < 0.005, and ∗∗∗∗*p* < 0.001.

**Figure 9:**
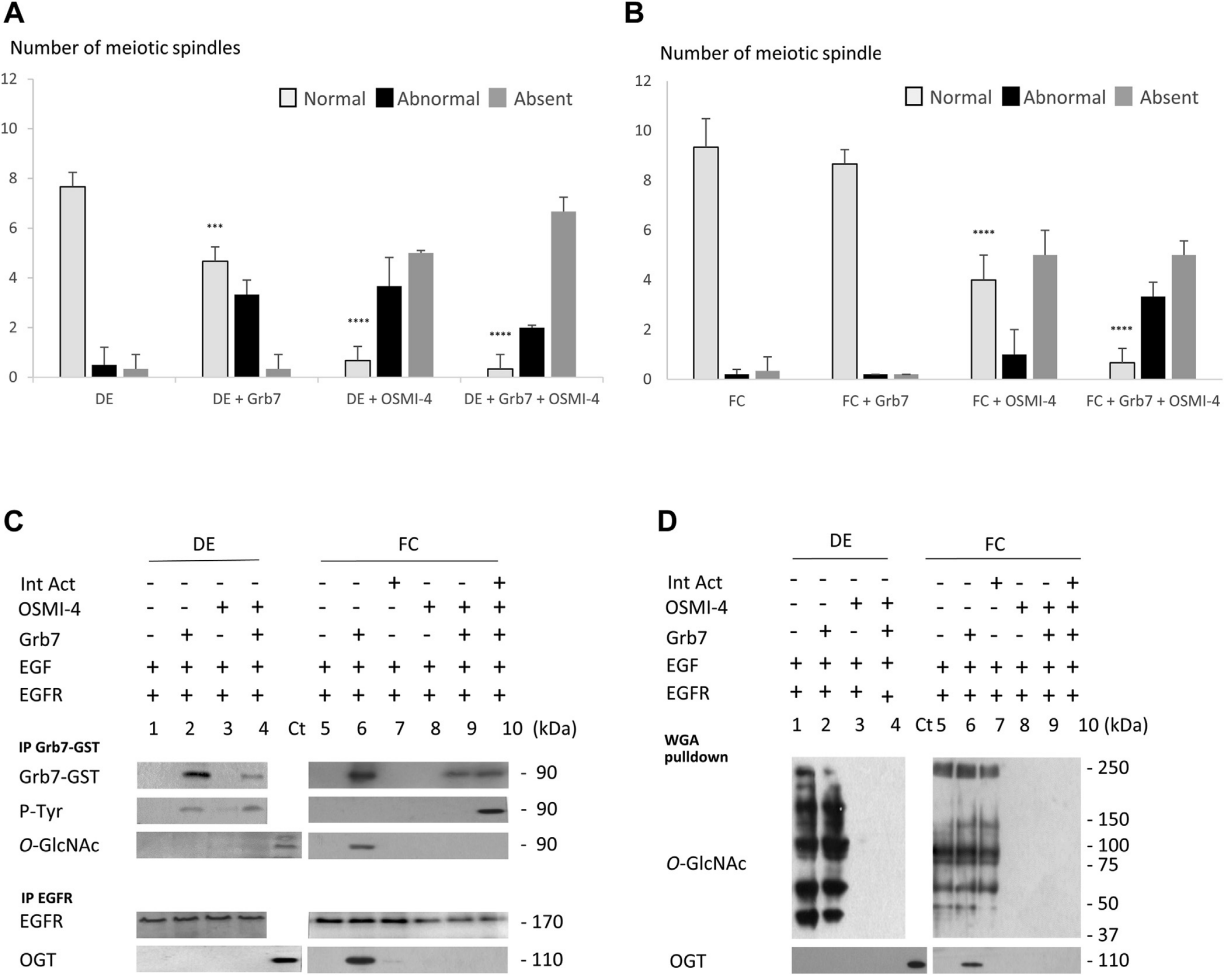
**Inhibition of *O*-GlcNAcylation using an *O-*GlcNAc transferase (OGT) inhibitor increases abnormal oocyte spindles.** Defolliculated (DE) and folliculated oocytes (FC) expressing epidermal growth factor (EGF) receptor (EGFR), microinjected with Grb7 (15ng) or not were microinjected or not with 15 μM OGT inhibitor OSMI-4, 1 h before EGF stimulation (5 nM). FCs were treated with or without 10 μM of pyrintegrin activator (Int Act). *A* and *B*, normal, abnormal, and absent spindles were scored on semithin sections (7 μm) stained with nuclear red and picroindigocarmine in oocytes. *C*, Grb7-GST or EGFR immunoprecipitations were realized and followed by Western blots with anti-*O*-GlcNAcylation (*O*-GlcNAc), anti-GST (Grb7-GST), and anti-OGT antibodies. *D*, sWGA pulldowns were followed by Western blots with anti-*O*-GlcNAcylation (*O*-GlcNAc) and anti-OGT antibodies. Experiments were repeated on 10 to 20 oocytes from two to three *Xenopus* females. Statistical significance against DE or FC was accepted for ∗∗∗*p* < 0.005 and ∗∗∗∗*p* < 0.001. GST, glutathione-*S*-transferase; sWGA, succinylated wheat germ agglutinin.

To understand the interplay between Grb7 *O*-GlcNAcylation and phosphorylation, experiments are performed with inhibitors targeting OGA or OGT, respectively, Thiamet G (Sigma–Aldrich; Figs. 6 and 7) and OSMI-4 (Figs. 8 and 9). Thiamet G at 50 μM could affect neither DE oocytes' nor FC oocytes’ GVBD percentage or GVBD_50_ significantly (Fig. 6, *B* and *D*). At 100 μM, Thiamet G induces a significant decrease in GVBD_50_ similar to a value observed with 15 ng of Grb7 in DE but not in FC (Fig. 6, *A* and *C*). In addition, abnormal spindle formation is scored in DE oocytes but not in FC oocytes (Fig. 7, *A* and *B*). About 100 μM of Thiamet G is further used as the lowest dose capable to reduce GVBD_50_ and to increase the formation of abnormal meiotic spindle in DE oocytes (Figs. 6*C* and 7*A*). The value of Gbr7 (15 ng) is chosen as the lowest value capable of significantly reducing the GVBD_50_ of the EGFR–EGF signaling from DE oocytes (Fig. 6*C*) and increasing abnormal meiotic spindle formation (Fig. 7*A*). The concomitant addition of Grb7 and Thiamet G lowers GVBD_50_ significantly compared with Grb7 or Thiamet G treatment alone and increases significantly the formation of abnormal spindles in DE oocytes (Figs. 6*C* and 7*A*), whereas no effects are observed in FC oocytes (Figs. 6*D* and 7*B*). Immunoprecipitations and sWGA pulldown to reveal this effect is associated with the recruitment of phosphorylated Grb7 without OGT in the EGFR complex in DE oocytes (Fig. 7, *C* and *D*). Grb7 phosphorylation is present in DE oocytes after Thiamet G treatment (Fig. 7, *C* and *D*). When the phosphorylation on Grb7 is prevented in DE oocytes (Fig. 7*C*), with the integrin inhibitor echistatin (500 nM), GVBD_50_ and the number of abnormal spindles are similar to those observed for Thiamet G alone (Figs. 6*C* and 7*A*). However, in those conditions in DE oocytes, Grb7 is not *O*-GlcNAcylated (Fig. 7, *C* and *D*). FC oocytes display no significative decrease in GVBD percentage, GVBD_50_, or normal spindle formation under Thiamet G addition (Figs. 6, *B* and *D* and 7*B*). Moreover, in FC oocytes treated with Thiamet G, Grb7 is *O*-GlcNAcylated in immunoprecipitated and sWGA pull-down experiments, and OGT is present in the EGFR complex (Fig. 7, *C* and *D*).

OSMI-4 effect was determined on the GVBD induced by EGFR–EGF in DE and FC oocytes expressing Grb7 (15 ng) after its microinjection. A concentration of OSMI-4 (30 μM) blocks the GVBD in DE and FC oocytes (Fig. 8, *A* and *B*). A lower concentration of OSMI-4 (15 μM) reduces the percentage of GVBD obtained in DE (to 50% compared with the control 83%) and FC oocytes (50% compared with the control 90%) (Fig. 8, *A* and *B*). The concentration of OSMI-4 (15 μM) is further used in the experiments as the lowest value capable to induce an effect. The combined action of Grb7 and OSMI-4 (in DE oocytes) decreases the GVBD (to 27%) compared with OSMI-4 alone (50%), whereas in FC oocytes, the GVBD percentage is unaffected (respectively, 53 and 57%) (Fig. 8, *A* and *B*). GVBD_50_ in DE and FC oocytes are not accelerated except for Grb7 in DE oocytes and Grb7–OSMI-4–integrin activator in FC oocytes (Fig. 8, *C* and *D*). DE oocytes treated with Grb7–OSMI-4 never reached GVBD_50_ (Fig. 8*C*). In DE oocytes submitted to Grb7, OSMI-4, or Grb7/OSMI-4 and in FC oocytes treated with OSMI-4 or Grb7–OSMI-4, the number of normal spindles is significantly lower (Fig. 9, *A* and *B*). In addition, in DE oocytes microinjected with Grb7 or Grb7–OSMI-4, Grb7 is phosphorylated, but not *O*-GlcNAcylated, and OGT is not present in the EGFR complex independently of OSMI-4 treatment (Fig. 9, *C* and *D*). In FC oocytes, Grb7 is *O*-GlcNAcylated in the control but not after OSMI-4 or after integrin activator treatments (Fig. 9, *C* and *D*). Moreover, Grb7 *O*-GlcNAcylation and OGT recruitment in the EGFR complex are lost after OSMI-4 and integrin activator treatments (Fig. 9, *C* and *D*). FC oocytes treated with Grb7–OSMI-4–integrin activator that display an accelerated GVBD_50_ also show a phosphorylated Grb7 (Fig. 9*D*).

## Discussion

Follicular cells around *Xenopus* oocyte exert multiple roles during oogenesis and could be involved in the regulation of oocyte signaling during meiosis (2, 47, 48). In the present work, we have investigated the role played by follicular cells on oocyte integrins. Immature stage VI oocytes blocked in prophase I express the insulin and the progesterone receptors without any other RTKs, and mRNA-encoding EGFR family members are only expressed during embryonic development (49). In mammals, EGFR is involved in the meiotic maturation process of the oocyte, during ovulation to retract and uncouple filopodia from the granulosa cells (43), but such a mechanism is not described for amphibians. In *Xenopus*, oocyte release from the ovarian follicles occurs synchronously with the GVBD requiring MAPK and matrix metalloproteases (7, 50). Oocyte heterologous expression of two residents of the integrin complex, EGFR, and Grb7, allowed us to investigate the relevance of the integrin signaling pathway with and without follicular cells (31, 32, 33, 51). Oocytes submitted to manual dissection and enzymatic treatment to remove their follicular cells remain unaltered (52) and retain their integrins.

In fully grown stage VI oocytes expressing EGFR and Grb7, stimulated by EGF, and deprotected from their surrounding follicular cells, the maturation from prophase I to metaphase II, also called GVBD, appears earlier compared with fully FCs (33, 53, 54, 55). In both deprotected and protected oocytes expressing EGFR–Grb7, the canonical tyrosine phosphorylation of the EGFR occurs (56, 57, 58, 59, 60). Integrin β1 and FAK-binding partners are anchored in the EGFR complex of deprotected oocytes, whereas Grb7 is bound to both deprotected and protected oocytes. In somatic cells, EGFR forms a complex with integrins (61), which recruits FAK usually localized to cellular contact points to enhance integrin signaling (61, 62, 63, 64). FAKs are present in oocytes of several species and activated before fertilization (65). In *X. laevis*, FAKs have been described (56, 57, 58). In our experiments using deprotected oocytes, Grb7 competitive peptides Y188 and Y338 or FAK antibodies are efficient counteracting tools showing both sites are involved in the mechanism of earlier GVBD_50_. These phosphorylated sites are in agreement with the FAK-targeted sites in Grb7 (37, 59). Our results imply that oocyte integrins’ capability to recruit FAK and form a heteromeric complex is downregulated in the presence of follicular cells. Follicular cells could modulate the formation of signaling complexes to avoid unwanted premature meiosis progression. The only RTK present in immature oocytes is the insulin–IGF1 receptor (3, 60), whereas the fibroblast growth factor receptor is translated during oocyte maturation (66). Both receptors interact with Grb7 (32), and it cannot be excluded such that heterocomplex formation would perturb normal meiosis.

A possible mutual exclusion between *O*-GlcNAcylation and phosphorylation is pointed out by our results. *O*-GlcNAcylation is known to compete and establish a dynamic interplay with other post-translational modifications, such as phosphorylation, on the same or adjacent sites (67). Amino acids phosphorylated or *O*-GlcNAcylated acting in a mutually exclusive manner are crucially involved in controlling protein activity (68). Formerly, *O*-GlcNAcylation was shown to act as a switch in the response ability of EGFR-mediated complex formation with integrin β1 (69), and the recruitment of OGT (responsible for *O*-GlcNAcylation) was described in an *O*-GlcNAcylated EGFR complex (70). In our experiments, Grb7, but not EGFR, is *O*-GlcNAcylated raising the possibility that the former is a substrate of OGT. A forced *O*-GlcNAcylation of Grb7 could induce the concomitant recruitment of OGT with Grb7 binding to the EGFR complex and in a reverse situation that forced Grb7 to gain phosphorylation and lose *O*-GlcNAcylation, OGT recruitment was lost in the EGFR complex.

Protein *O*-GlcNAcylation is mandatory for *Xenopus* oocyte maturation. *O*-GlcNAcylation was formerly shown pivotal for progesterone-induced maturation in naïve oocytes (25, 27). In our experiments, GVBD percentages are decreased with a microinjected low concentration of OGT inhibitor and abolished with higher doses in both DEs and FCs. Moreover, oocytes expressing EGFR–Grb7 unshielded from their follicular cells are sensitive to OGA inhibition resulting in a significantly earlier GVBD and an abnormal spindle formation. In these DE oocytes, the increased *O*-GlcNAcylation that accelerates the occurrence of the GVBD_50_ is additive to the effect produced by Grb7 phosphorylation. The GVBD acceleration under OGA inhibition agrees with former results obtained on DE progesterone-stimulated oocytes where *O*-GlcNAcylation was experimentally increased by OGT microinjection (26).

In most metazoans, centrioles are eliminated (71), and chromosomal transmission at meiosis relies on acentrosomal spindle assembly (72, 73, 74). During prophase I to metaphase II meiosis transition, *Xenopus* oocytes' bipolar acentriolar spindle rotates and anchors beneath the plasma membrane at one edge of the animal pole (52). In somatic cells, integrin β1 activation plays a critical role in the control of spindle orientation (75, 76, 77). No such evidence exists in *Xenopus* oocyte. However, we have shown that in deprotected oocytes, integrin activation and complex formation with EGFR–Grb7 deregulate these processes necessary to anchor the spindle to the plasma membrane showing that integrin signaling can interfere with meiosis spindle formation. Meiosis spindle regulation probably depends on the coordinated addition and removal of *O*-GlcNAc moieties. Abnormal spindle formation was observed in oocytes with early occurring GVBD either under treatment with *O*-GlcNAcylation modulators or by the activation of the integrin–EGFR–Grb7 complex. The *O*-GlcNAcylation of many structural proteins such as actin and tubulin is necessary for *Xenopus* oocyte to undergo progesterone-induced maturation, and OGT has been localized on the meiotic spindle (51). In somatic cells, while it differs from the meiotic spindle being acentriolar, the organization of the mitotic spindle relies on *O*-GlcNAc cycling (78, 79).

**Figure 10:**
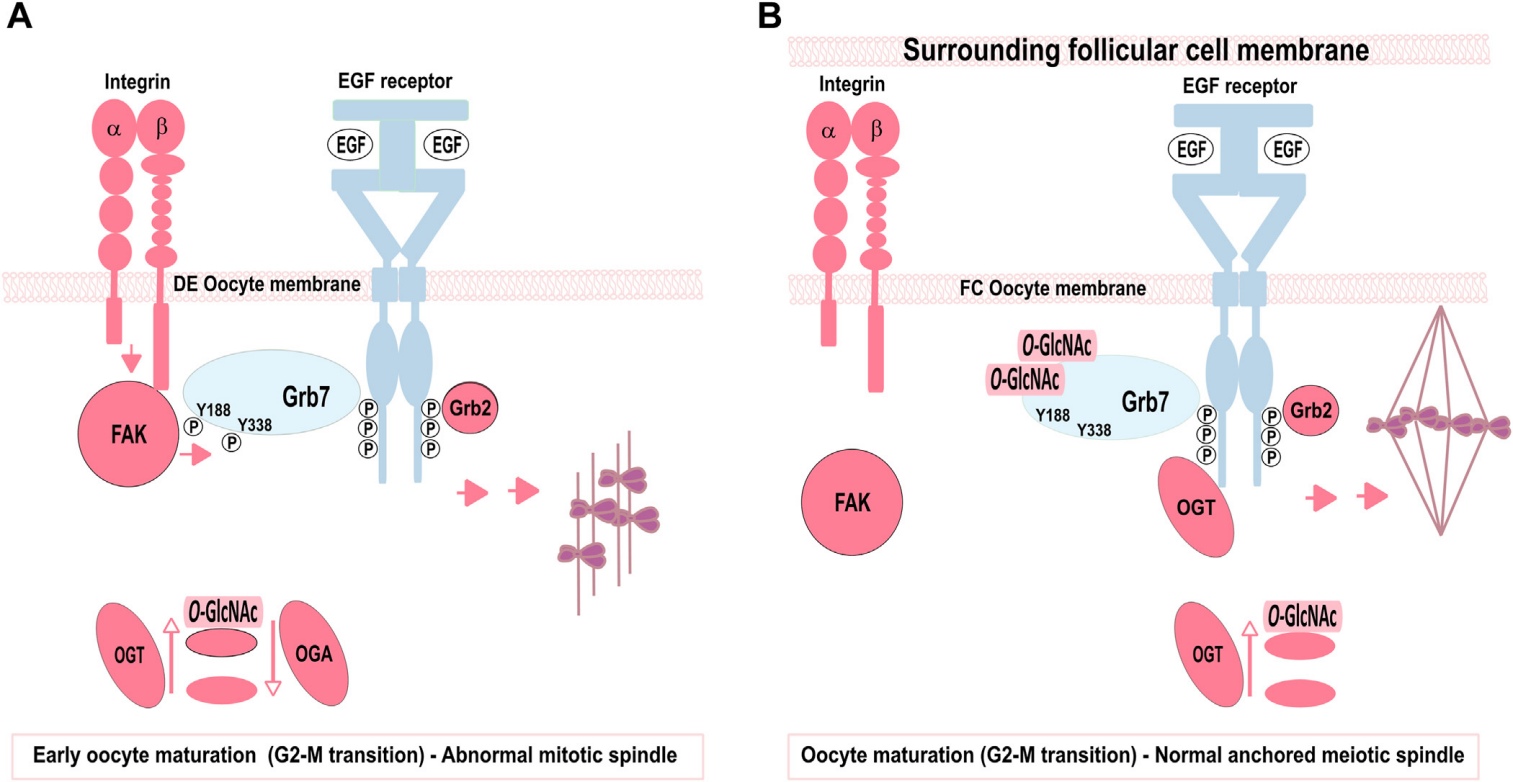
**Model for *Xenopus* oocyte protection by follicular cells from abnormal maturation (G2–M transition) triggered by an epidermal growth factor (EGF) receptor (EGFR)/integrin–FAK complex and the adaptor Grb7 phosphorylation.***A*, in defolliculated oocytes (DE), EGF addition to EGFR-expressing oocytes triggers the tyrosine kinase receptors autophosphorylation (P) and the recruitment of effectors including adaptors Grb2 and Grb7. Activated EGFRs form heterocomplexes with integrin–FAK, leading to Grb7 phosphorylation on two tyrosine residues Y188 and Y338. Consequently, these signaling pathways favor a rapid meiotic transition from prophase I to metaphase II (maturation) and abnormal spindle formation that does not localize at the oocyte plasma membrane. *B*, in follicular cells (FCs), the formation of a multicomplex between EGFR–EGF and integrin–FAK is prevented by the layer of surrounding follicular cells and their tight contact with the oocyte membrane. Phosphorylated EGFRs recruit OGT, Grb7 is *O*-GlcNAcylated, and abnormal oocyte phenotype is avoided. The oocyte OGT–OGA balance avoids unwanted phenotypic defaults. In *A* and *B*, inhibition of protein *O*-GlcNAcylation (OGT inhibitor) increases phenotypic defaults. Folliculated oocytes are more insensitive toward protein *O*-GlcNAcylation removal (OGA inhibition). Oocyte endogenous proteins are in *red*, and heterologous expressed proteins are in *blue*. FAK, focal adhesion kinase; OGT, *O-*GlcNAc transferase.

In conclusion, follicular cells protect *Xenopus* oocytes from early unwanted GVBD associated with abnormal spindle formation by avoiding integrin–FAK signaling activation and inducing a multimeric complex with heterologously expressed EGFR–Grb7. Follicular-surrounding cells act as negative regulators on oocyte integrin β1 to allow anchorage of OGT in the integrin–EGFR–Grb7 complex and favor the *O*-GlcNAcylation of Grb7. A model is proposed in Figure 10. These evidences reinforce the protective role played by follicular cells to avoid unexpected early signaling and deleterious spindles. In oocytes surrounded by their follicular cells, OGT allows normal spindle formation. Our results also unravel a finely orchestrated regulation between OGT and OGA during meiosis progression.

## Experimental procedures

Reagent-grade chemicals were purchased from Sigma–Aldrich unless specified.

### Animal and oocyte handling

All animal experiments were performed according to the rules of the European Union Directive for laboratory animal experimentation 2010/63/EU. The protocol of this study was approved by the local institution (Comité d’Ethique en Expérimentation Animale, Haut de France, G59-00913). Mature *X. laevis* females, purchased from the CRB-University of Rennes I, and housed in PHExMAR, at the University of Lille, were anesthetized by immersion in 1 g/l MS222 solution (tricaine methane sulfonate). After anesthesia, ovarian lobes were surgically removed and stored in ND96 medium (96 mM NaCl, 2 mM KCl, 1.8 mM CaCl_2_, 1 mM MgCl_2_, 5 mM Hepes–NaOH, pH 7.5) at 14 °C until required.

### Follicular cell removal, oocyte microinjection, and treatments

To remove the layer of follicular cells, stage VI oocytes were defolliculated by partial ovarian digestion with collagenase A treatment for 30 min (1 mg/ml) followed by manual microdissection under a stereomicroscope. For oocytes that remained surrounded by their follicular cells, the outer layer was locally pinched opened manually in the equatorial region using a watchmaker's forceps (Moria) to reveal a tiny area of the plasma membrane to allow further microinjection. Sixty nanograms of mRNA of the human EGFR (33) were microinjected in the equatorial region of the oocytes using a positive displacement digital micropipette (Nichiryo). FC and DE oocytes were incubated at 19 °C in ND96 medium for 2 days before they were submitted to a second microinjection or not with Grb7 (5, 10, 20, and 100 ng), 40 nl of an integrin β1 antibody (M-106; Santa Cruz Biotechnology) (80), 60 ng of SH2 domain of Grb7 (32) for 30 min, before they were treated or not with 500 nM of an RGD-dependent integrin inhibitor for α5β1, αvβ3, echistatin (81, 82), 10 μM of pyrintegrin activator (Calbiochem) (83), 50 to 100 μM of Thiamet G, or microinjected with 15 or 30 μM OSMI-4 (Medchemexpress), competing peptides KNFAKY(188)ELFKQ, FRLFKY(338)GVQL (30 ng/30 nl), and mismatched peptide KELVGYRFLQ (NeoSystems). One hour later, oocytes were stimulated by 5 nM of human EGF and stored at 19 °C. Isolation of follicular cells was realized manually with forceps under a stereomicroscope.

### Preparation of RNA and fusion protein

The human EGFR pOBER vecto was linearized with NotI, and capped cRNA was transcribed using SP6 RNA polymerase (mMESSAGE mMACHINE kit; Ambion) (32, 33). Grb7 adapter and SH2 domain of Grb7 were produced as GST fusions as described (32, 84).

### Meiotic resumption analysis

Oocytes displaying a white spot, a rise of the germinal vesicle at the animal pole reflecting meiotic progression from prophase I to metaphase II, were individually scored 12 h after EGF stimulation (85, 86). The time required to obtain 50% of mature oocytes (GVBD_50_) was determined to enable the comparison between the maturation kinetics of 10 to 20 oocytes from three different females in each experiment. In the case of the phenotypic absence of the white spot, oocytes were heat fixed for 15 min at 100 °C and bisected along the animal–vegetative axis to ascertain the presence of the germinal vesicle. Statistical significances (mean ± SD) were determined by two-way ANOVA followed by Dunnett’s multiple comparison tests (∗*p* < 0.05, ∗∗*p* < 0.01, ∗∗∗*p* < 0.005, and ∗∗∗∗*p* < 0.001).

### Electrophoresis and Western blot

Oocytes were lysed in the following PYT homogenization buffer: 50 mM Hepes (pH 7.4), 500 mM NaCl, 0.05% (m/v) SDS, 0.5% (v/v) Triton X-100, 5 mM MgCl_2_, 1 mg/ml bovine serum albumin, 10 μg/ml leupeptin, 10 μg/ml aprotinin, 10 μg/ml soybean trypsin inhibitor, 10 μg/ml benzamidine, 1 mM PMSF, 1 mM sodium vanadate, with a ratio of 10 oocytes/100 μl of buffer. After a 12,000*g* centrifugation for 10 min at 4 °C, the supernatant was denatured in 2× Laemmli buffer (65.8 mM Tris–HCl [pH 6.8], 26.3% [v/v] glycerol, 2.1% [m/v] SDS, 0.01% [m/v] bromophenol blue, and 4% [v/v] β-mercaptoethanol; Bio-Rad) at 96 °C for 3 min. Proteins were separated by 4 to 20% SDS-PAGE gels (mini protean TGX; Bio-Rad) for 1 h at 200 V in denaturing buffer (0.1% [m/v] SDS, 0.3% [m/v] Tris base, and 1.44% glycine) and transferred onto nitrocellulose membrane (Amersham Hybond) by wet transfer (0.32% [m/v] Tris, 1.8% [m/v] glycine, and 20% [v:v] methanol) for 1 h at 100 V. Membranes were blocked with 5% (m/v) low fat dry milk or bovine serum albumin in Tris-buffered saline (TBS) added with 0.05% (v/v) Tween and incubated overnight at 4 °C with specific primary antibodies: rabbit polyclonal antibodies were raised against integrin β1 (M-106; Santa Cruz Biotechnology; 1/1500 dilution), OGT (DM17; Sigma–Aldrich; 1/1000 dilution); mouse monoclonal antibodies were raised against EGFR (Santa Cruz Biotechnology; 1/1000 dilution), FAK (2A7; Sigma–Aldrich; 1/1000 dilution), GST (B-14; Santa Cruz Biotechnology; 1/1500 dilution), phosphotyrosine (PY20; from Sigma–Aldrich; 1/1000 dilution), *O*-GlcNAc (RL-2; Thermo Fisher Scientific; 1/1200 dilution), and goat polyclonal antibody was raised against β-actin (Santa Cruz Biotechnology; 1/1200 dilution). After three washes of 10 min each in TBS–Tween, nitrocellulose membranes were incubated for 1 h with the appropriate horseradish peroxidase–labeled secondary antibodies: anti-rabbit or antimouse antibodies (Invitrogen, by Thermo Fisher Scientific Biosciences GMBH; 1/30,000 dilution) or antigoat antibodies (Santa Cruz Biotechnology; 1/30,000 dilution). After three washes in TBS–Tween for 10 min each, the signals were revealed with a chemiluminescent assay (ECL Select; GE Healthcare) on hyperfilms (Amersham hyperfilm MP). Follicular cells were removed manually from 30 oocytes and used for Western blot after homogenization in PYT buffer using a grinding pestle (1.5 ml tube). After centrifugation at 12,000*g* for 10 min, the concentration of the supernatants was evaluated using the Bradford assay (Bio-Rad) at 595 nm (SPECTROstar Nano; BMG LABTECH). Three independent experiments were performed on three different females.

### Coimmunoprecipitations

Oocyte lysates were obtained using PYT buffer as described in the Western blot section. After a 12,000*g* centrifugation for 10 min at 4 °C, pellets were resuspended and homogenized using Eppendorf micropestles (SA). After a 12,000*g* centrifugation for 10 min at 4 °C, lysates were precleared at 4 °C for 1 h with Protein A Sepharose (20 μl of 50% beads/200 μl of cell lysate; from Sigma–Aldrich) under gentle rocking. After brief centrifugation, supernatants were incubated at 4 °C for 1 h with antibodies raised against either EGFR (1/100), GST (1/100), mouse IgG (1/100), or rabbit IgG (1/100) under rotation and followed by incubation with Protein A Sepharose (20 μl of 50% bead slurry) for 1 h at 4 °C under rotation. After three washes with PYT buffer without detergent, the pellets were collected by brief centrifugation, resuspended in 2× Laemmli buffer, and heated at 96 °C for 3 min before SDS-PAGE and Western blots were performed.

### sWGA pull down

Twenty oocytes were lysed in 200 μl of radioimmunoprecipitation assay buffer (50 mM Tris [pH 7.4], 150 mM NaCl, 5 mM EDTA, 1% Triton X-100, 2% NP-40, 0.1% SDS, and10 μg/ml leupeptin, 10 μg/ml aprotinin, 10 μg/ml soybean trypsin inhibitor, 10 μg/ml benzamidine, 1 mM PMSF, and 1 mM sodium vanadate) as described (26). After a centrifugation at 12,000*g* for 10 min at 4 °C, lysates were incubated with 50 μl of sWGA agarose beads (Vector Laboratories) for 2 h at 4 °C or with sWGA beads preincubated with 0.5 M free GlcNAc (TCI) for 1 h, to control the specificity of the reaction. sWGA-bound proteins were washed four times and eluted from the beads in 2× Laemmli buffer before SDS-PAGE was performed.

### Cytological analysis

Abnormal spindles were detected as described previously (49, 50). Oocytes were fixed in Smith reagent (Smith A: potassium bichromate [17 mM]; Smith B: formic acid and acetic acid 80/20%, [m/v]) for at least 12 h before they were dehydrated and embedded in paraffin. Sections of 7 microns were cut and stained with picroindigocarmine (0.25 g of picroindigocarmine QSP 100 ml saturated picric acid) to reveal cytoplasmic structures and nuclear red (0.1 g of nuclear red QSP 100 ml aluminum sulfate 5% [m/v]) for nuclear structures and chromosomes. For oocyte hemisections, oocytes were fixed in paraformaldehyde 8% (m/v) at 4 °C at least overnight in ND96 medium before they were briefly rinsed and sectioned using a fine razor blade along the animal–vegetative axis. For nuclear staining, oocytes defolliculated or not, expressing EGFR for 12 h and microinjected with Grb7, were incubated 15 min at room temperature with Hoechst 33258 at 1 μg/ml final concentration in ND96 medium. After three rinses, images were captured under a confocal microscope *Nikon A1R* (TISBIO).

### Statistics

Statistical significances (mean ± SD) were determined by two-way ANOVA followed by Dunnett’s multiple comparison tests (∗*p* < 0.05, ∗∗*p* < 0.01, ∗∗∗*p* < 0.005, and ∗∗∗∗*p* < 0.001).

## Data availability

All data are contained within the article.

## Supporting information

This article contains supporting information (Figure S1).

## Conflict of interest

The authors declare that they have no conflicts of interest with the contents of this article.
