# Supplementary material for: Follicular cells protect Xenopus oocyte from abnormal maturation via integrin signaling downregulation and O-GlcNAcylation control
Source: J Biol Chem. 2023 Jun 23;299(8):104950. doi: 10.1016/j.jbc.2023.104950 (PMC10366548; doi:10.1016/j.jbc.2023.104950)
Supplement: Supporting figure S1 [file mmc1.docx]

**Supporting information**

**Follicular cells protect *Xenopus* oocyte from abnormal maturation *via* integrin signaling downregulation and *O*-GlcNAcylation control**

Alain Martoriati^1¤^, Caroline Molinaro^1¤^, Guillaume Marchand^1^, Ingrid Fliniaux^1^, Matthieu Marin^1^, Jean-François Bodart^1^, Yoshiko Takeda-Uchimura^1^, Tony Lefebvre^1^, Vanessa Dehennaut^2^, Katia Cailliau^1*^

**
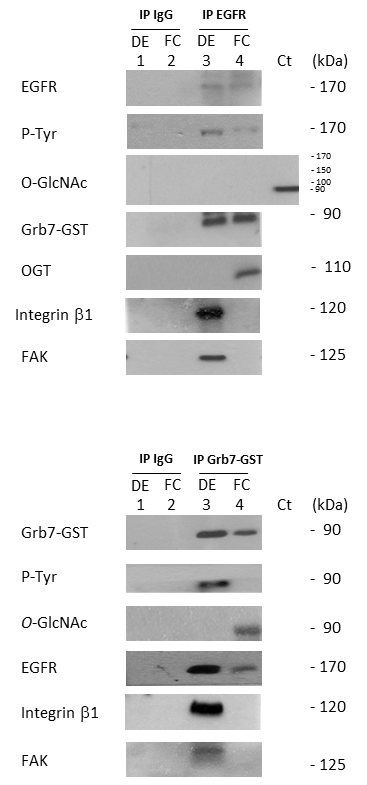
**

**Figure S1.** Stage VI *Xenopus* oocytes surrounded by follicular cells (FC) or defolliculated (DE) and expressing EGFR were injected with 20 ng of Grb7 for 1 h and stimulated by EGF (5nM). Immunoprecipitations were performed with anti-EGFR, anti-GST-Grb7, or IgG antibodies before Western blots were performed using antibodies against EGFR, phosphotyrosine (P-Tyr), *O*-GlcNAcylation (*O*-GlcNAc), GST (Grb7-GST), OGT, Integrin β1, and FAK. Control (Ct) was an immunoprecipitation performed with anti-GST-Grb7 on FC cells expressing EGFR and injected with Grb7.
